# Chronic Recurrent Multifocal Osteomyelitis in Pediatric Crohn Disease, A Paradoxical Effect to Antitumor Necrosis Factor Alpha

**DOI:** 10.1097/PG9.0000000000000007

**Published:** 2020-08-20

**Authors:** Anne Cordesse, Emmanuelle Ecochard-Dugelay, Isabelle Melki, Marion Caseris, Nadia Belarbi, Jean-Pierre Hugot, Jerome Viala, Christine Martinez-Vinson

**Affiliations:** From the * Service de Nutrition et Gastroentérologie Pédiatriques, APHP, Hôpital Trousseau, France; † Service de Gastroentérologie et Nutrition Pédiatriques, APHP, Hôpital Robert Debré, France; ‡ Service de pédiatrie générale, maladies infectieuses et médecine interne pédiatrique, APHP, Hôpital Robert Debré, France; § Service d’imagerie pédiatrique et fœtale, APHP, Hôpital Robert Debré, France; ¶ Université Paris Diderot, Paris, France; ∥ INSERM UMR 1123 (ECEVE), Paris, France.

**Keywords:** Crohn disease, anti-TNF-α, chronic recurrent multifocal osteomyelitis, paradoxical effect

## Abstract

Tumor necrosis factor-α (TNF-α) inhibitors have resulted in significant progress in the treatment of chronic inflammatory diseases. However, these therapies can lead to paradoxical immune-mediated inflammatory diseases with unknown physiopathology. For the first time, we report 3 cases of paradoxical chronic recurrent multifocal osteomyelitis after infliximab or adalimumab therapy during the course of Crohn disease. The patients complained of bone pain without joint involvement. At the time of diagnosis of paradoxical reaction, all patients were in remission due to anti-TNFα efficiency. Trough levels of anti-TNFα were in the expected range, and there were no anti–anti-TNFα antibodies. The duration of treatment was between 2 and 26 months. Other causes of CRMO were excluded. All patients recovered after discontinuation of infliximab (n = 2) or adalimumab (n = 1). The increasing use of these therapies leads to new descriptions of paradoxical effects, which clinicians should be aware of.

Over the last 2 decades, anti–tumor necrosis factor-α (TNFα) agents have been increasingly used to induce and maintain clinical remission in patients with Crohn disease (CD) (1–4). Nevertheless, anti-TNFα has been associated to paradoxical immune-mediated inflammatory diseases that can be defined as the appearance or exacerbation of a pathological condition that usually responds to this class of drug while treating a patient for another condition (5–7). Infrequent and probably underreported, the physiopathology of these paradoxical effects remains unknown and may implicate an imbalance of cytokines (5–7). For the first time, we report 3 cases of paradoxical chronic recurrent multifocal osteomyelitis (CRMO) after anti-TNFα therapy during the course of CD in 3 children.

Informed patient consent was obtained for publication of these case details.

## CASE 1

A 7-year-old girl was admitted to our department with a typical history of CD. The endoscopy test result showed a pancolitis sparing the rectum. Infliximab was initiated 1 month later for perianal lesions, allowing resolution of gastrointestinal symptoms. However, her symptoms relapsed 17 months later. The girl stopped infliximab and was treated with adalimumab associated to methotrexate, which was transiently efficient for a year, but the symptoms relapsed afterwards. The clinician decided to switch to tacrolimus. Five months later, as the disease was insufficiently controlled, treatment with infliximab was proposed again (10 mg/kg every 5 weeks), allowing for a new remission. Trough levels to infliximab were 2.3 μg/ml, and no antibodies to infliximab were found.

**FIGURE 1.**
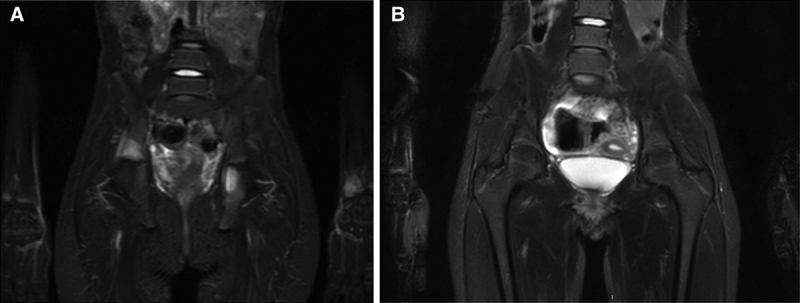
Whole-body magnetic resonance imaging (MRI) with coronal short TI inversion recovery (STIR) sequences (case 1). A, Increased signal intensity of sacroiliac bones and left radial metaphysis at chronic recurrent multifocal osteomyelitis (CRMO) onset. B, Radiological control 3 years later: disappearance of these hypersignals.

Five months later, she presented with psoriasis skin lesions, coccyx and inguinal pain, and limping, with no history of trauma or infection. Inflammatory markers were normal. Magnetic resonance imaging (MRI) identified bone marrow edema of pelvis, sacroiliac joint, and left radial metaphysis (Fig. 1A). Infliximab was discontinued, and she received one subcutaneous ustekinumab injection. Clinical signs worsened, with fever and inability to walk, leading to hospitalization within 2 days. Inflammatory markers were elevated; no germs were isolated. A positron emission tomography/computed tomography revealed osteolysis lesions (spine, pelvis, radius, and right maxillary) and bilateral pulmonary nodules. Bone marrow aspiration was found to be normal. Pulmonary biopsy and bronchoalveolar lavage eliminated a tumoral or an infectious process. A primary immunodeficiency disease was ruled out. Radius bone biopsy showed severe inflammation, with a predominance of neutrophils and granulomatous reaction. A skin biopsy was in favor of psoriasis. A multidisciplinary medical team meeting argued for CRMO lesions. At a 2-year follow-up, she was in remission for both CD and CRMO under adalimumab (Fig. 1B).

## CASE 2

A 12-year-old girl was admitted to our department with a typical history of CD. The endoscopy showed ulceration of the terminal ileum and stricture of the ileocecal valve. Infliximab was initiated. Resolution of the gastrointestinal features was achieved within weeks with a 10 mg/kg every 6 weeks of infliximab dosing, trough levels to infliximab were 14.5 μg/ml, and no antibodies to infliximab were detected.

Two years later, she complained of isolated hip pain. MRI of the sacroiliac joints was normal. Two months later, she presented with right clavicle pain without fever. The sternoclavicular joint was swollen, without warmth or redness. Laboratory tests and X-ray performed were unremarkable. Infliximab was maintained, and the symptoms disappeared within 1 month.

**FIGURE 2.**
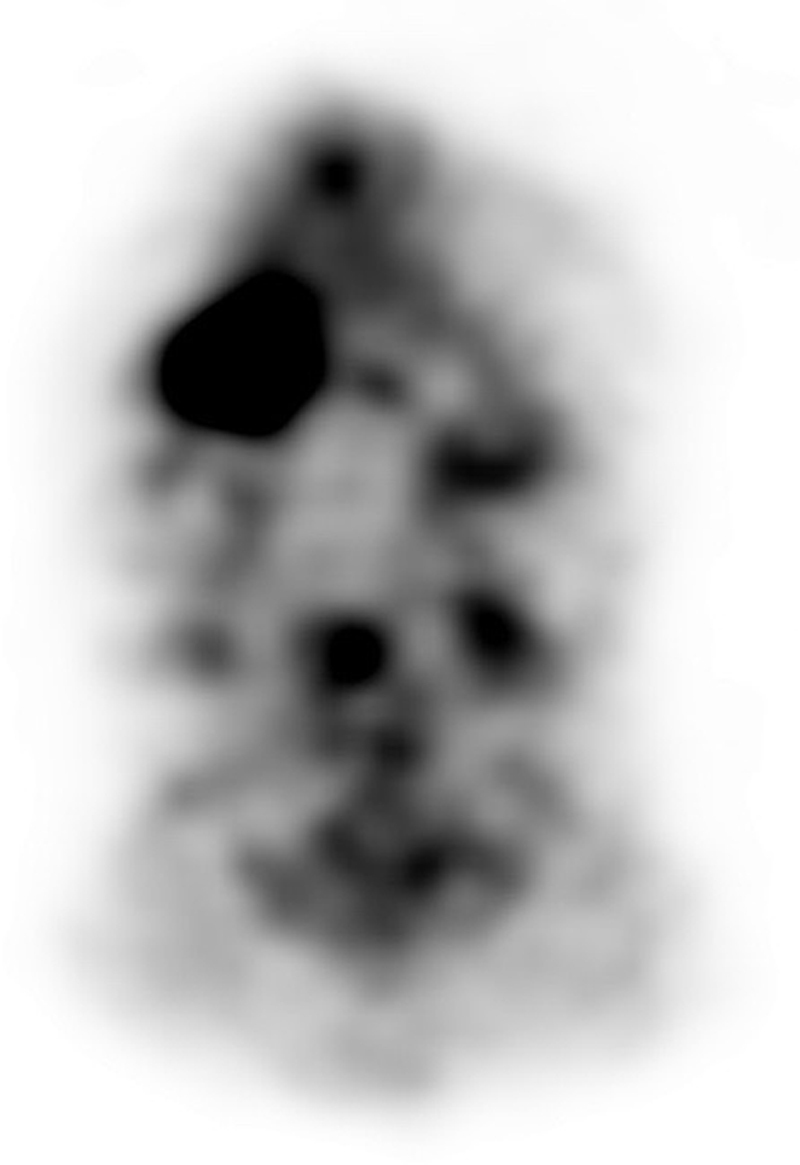
Whole-body magnetic resonance imaging (MRI) with coronal short TI inversion recovery (STIR) sequences (case 2). A, Increased signal intensity of thoracic and lumbar vertebral bodies, spinous processes, and right cotyl. B and C, Radiological control 6 months later: disappearance of these hypersignals.

Two months later, she suffered from moderate mechanical lower back pain associated with psoriasis of the scalp, with no gastrointestinal disorders. Inflammatory markers were normal. MRI of the spine revealed abnormal signals (low signal on T1, high on T2, and short TI inversion recovery) of thoracic and lumbar vertebral bodies and spinous processes. There was no evidence of an infectious process. Despite proper pain management, her symptoms worsened. Whole-body MRI was highly suggestive of CRMO lesions: same abnormal signals of thoracic and lumbar vertebral bodies and spinous processes, right cotyle, and metaphysis of proximal and distal tibia (Fig. 2A). At that point, CRMO was suspected as a paradoxical immune effect of infliximab. Anti-TNFα was suspended, and azathioprine was started. At a 6-month follow-up, whole-body MRI showed a decrease in skeletal lesions (Fig. 2B, 2C). CD was controlled with azathioprine alone.

## CASE 3

An 8-year-old girl was admitted for bloody diarrhea and failure to thrive. The endoscopy showed pancolitis. Infliximab was started 1 month after the diagnosis of CD because of steroid resistance and of acute severe colitis. After 4 infusions, and because of loss of response, adalimumab was started (40 mg every other week, trough levels to adalimumab were 7.8 μg/ml, and no antibodies to adalimumab were found). Twenty-two months later, the patient complained of lumbar and sciatic pain without fever. At the same time, ankle skin psoriatic lesions were observed and confirmed by dermatologists. Inflammatory markers were normal. A positron emission tomography/computed tomography and whole-body MRI revealed osteolytic lesions of spine, pelvis, and long bones. The sacral bone biopsy showed a granulomatous reaction. There was no argument for an infectious or a tumoral process. Adalimumab was disrupted and ustekinumab, a new therapeutic class, was initiated. Two months later, the patient recovered without any bone pain or psoriasis injury.

## DISCUSSION

Because osteoarticular manifestations are the most frequent extraintestinal manifestation of CD, CRMO diagnosis is not straightforward and requires experimented clinicians to avoid confusion with other differential diagnosis (8–10).

As CD has already been associated with CRMO, several arguments were necessary to suspect an anti-TNFα paradoxical event (11). The chronological association between the withdrawal of anti-TNFα and clinical improvement is suggestive of a paradoxical effect. Moreover, our cases shared similitudes with paradoxical psoriasis eruption: onset of CRMO lesions have been related to periods of CD remission; our patients had never reported clinical signs associated to CRMO before diagnosis, and they reported symptoms of CRMO several months after starting anti-TNFα. Furthermore, trough levels of infliximab were in the expected ranges, without any antibodies detected in the serum (5,12–14). CRMO signs appeared later than the median age of isolated CRMO (9). Finally, psoriasiform lesions following TNFα treatment were diagnosed in our 3 patients, which disappeared after stopping the incriminated drug, arguing for a second paradoxical disease (5,6).

Actually, the pathophysiology of these paradoxical effects is still unknown. Full blockage of TNFα in quiescent tissues may increase inflammatory pathways, negatively regulated by TNFα in normal conditions, leading to paradoxical reactions (6,15,16).

## ACKNOWLEDGMENTS

A.C., E.D., I.M., J.P.H., J.V., and C.M.V. worked on the conception and design of the project. A.C., E.D., I.M., N.B., J.P.H., J.V., and C.M.V. were involved in data acquisition. All authors contributed to the interpretation of results and writing. All authors approved the final version of the article.
